# A chromosome-scale nuclear genome and complete mitogenome of the bio-control fungus *Cordyceps cateniannulata*

**DOI:** 10.1038/s41597-026-07231-1

**Published:** 2026-04-14

**Authors:** Min Liu, Qiaosun Huang, Huiyu Tang, Ziqiang Wang, Wei Zheng, Zihao Wang, Xi Huang, Yixi Zhang, Yanan Wang, Luodong Huang

**Affiliations:** 1 College of Life Science and Technology, Guangxi University, Nanning, 530004 China; 2 State Key Laboratory for Quality Ensurance and Sustainable Use of Dao-Di Herbs, National Resource Center for Chinese Materia Medica, China Academy of Chinese Medical Sciences, Beijing, 100700 China

**Keywords:** Genome assembly algorithms, Fungal genomics

## Abstract

*Cordyceps cateniannulata*, a recently characterized entomopathogenic fungus, has been employed in biological control, plant disease management, and growth promotion. In this study, the complete genomic sequences of *C. cateniannulata* were generated using BGI, PacBio, and Hi-C sequencing platforms. The nuclear genome spans 32.71 Mb, comprising seven pseudochromosomes (chromosome-scale scaffolds) with an N50 length of 4,790,175 bp and a guanine–cytosine content of 53.82%. For the first time, a fully assembled mitochondrial genome of 24,712 bp and a ribosomal DNA tandem repeat sequence of 7,974 bp were obtained. Furthermore, after meticulous annotation and manual refinement of structural features, 10,744 protein-coding genes and 156 non-coding RNAs were identified. This study has produced the high-quality whole-genome map of the *C. cateniannulata*, offering substantial theoretical significance and application potential for the innovative exploitation of *Cordyceps* fungal germplasm resources. Additionally, it provides novel perspectives on the evolutionary trajectory, infection strategies, and ecological adaptability of entomopathogenic fungi.

## Background & Summary

In recent years, whole genome sequencing (WGS) has been extensively applied to investigate the associations between phenotypic traits and genetic mechanisms in entomopathogenic fungi. Lee *et al*. (2018) performed WGS on the biopesticide entomopathogenic fungus *Beauveria bassiana* strain JEF-007, producing a scaffold-level genome^1^. Comparative genomic analysis demonstrated that chitinase and trypsin-like protease genes within the JEF-007 genome were highly conserved across the species and played pivotal roles in regulating infection processes. *Metarhizium robertsii* strain DSM 1490 is capable of infecting a wide range of insects, including termites. Genomic analysis indicated that its genome spans 45.68 Mb, implying the existence of a complex infection system^2^.

*Cordyceps*, the type genus of *Cordycipitaceae*, constitutes the most diverse and widely distributed lineage of entomopathogenic fungi within *Hypocreales*. It encompasses a broad spectrum of bioactive compounds with recognized benefits to human health, rendering it highly valuable for applications in food, pharmaceuticals, and biological control^3^. Nevertheless, as of September 2025, the National Center for Biotechnology Information (NCBI) database records genomic data for only 65 strains representing 10 species of *Cordycipitaceae*, with merely three species assembled at the chromosome level. Additionally, the predicted genesets display deficiencies in structural integrity, boundary definition, and overall completeness, thereby impeding investigations of genetic evolution and metabolic functions in the genus *Cordyceps*. Therefore, enhancement of genome assembly quality and gene set annotation, with improved chromosome-level continuity and completeness, is imperative for enabling more comprehensive functional genomic analyses.

*Cordyceps cateniannulata* (Z.Q. Liang) Kepler, B. Shrestha & Spatafora is an entomopathogenic fungus classified within the order *Hypocreales*, family *Cordycipitaceae*, and genus *Cordyceps*^4^. As a dominant insect-parasitic species in forest ecosystems, it ranks second only to *B. bassiana* in abundance and exhibits a broad distribution across Brazil, China, Colombia, Japan, and Ethiopia^5^. Previous studies have demonstrated that *C. cateniannulata* possesses substantial potential for biological control, as it secretes diverse insecticidal metabolites such as cinerarin I^6^, which exhibits pronounced toxicity against arthropod pests from the orders *Lepidoptera*, *Hemiptera*, *Coleoptera*, and *Acari*. Montes-Bazurto *et al*. (2020) reported that *C. cateniannulata* effectively suppressed populations of the defoliating pest *Stenoma impressella* in Colombian oil palm plantations^7^. Additionally, this fungus was shown to cause significant infection and mortality in larvae of *Allantus luctifer*, a pest of buckwheat, representing the first documented case of fungal control against *A. luctifer*^8^. Recently, Pereira *et al*. (2024) demonstrated that *C. cateniannulata* could efficiently inhibit the causal agent of coffee leaf rust, thereby mitigating the severe yield losses associated with *Hemileia vastatrix* outbreaks in coffee-producing regions^5^. Moreover, research has indicated that *C. cateniannulata* contributes to plant growth promotion and enhances resistance to both insect infestation and disease through root colonization^9,10^.

*C. cateniannulata*, noted for its considerable potential in biological control, has emerged as a focal point of research in recent years, necessitating high-quality genomic resources to further clarify its ecological adaptability and the molecular genetic mechanisms underlying insect infection. To bridge this gap, the complete genomic dataset of *C. cateniannulata* strain GXU-8616 parasitizing Lepidopteran pupae was obtained using a hybrid sequencing strategy that integrated MGISEQ-200, PacBio HiFi, and Hi-C technologies, producing a chromosome-level nuclear genome of high quality, a fully assembled circular mitochondrial genome, and tandemly repeated complete ribosomal sequences.

The nuclear genome of GXU-8616 was determined to span 32.71 Mb, of which 2.44% consisted of repetitive sequences. Hi-C scaffolding yielded seven pseudochromosomes, with an N50 length of 4,790,175 bp, a guanine-cytosine (GC) content of 53.82%, and a benchmarking universal single-copy orthologs (BUSCO) analysis indicating 99.0% completeness (fungi_odb10 database). Through the integration of ab initio prediction, homology- and transcript-based approaches, combined with manual refinement using IGV-Gsaman and transcriptome data, 10,744 genes were annotated, all with intact boundary sequences, and BUSCO analysis confirmed gene set completeness of 99.6% (fungi_odb10 database). Additionally, a mitochondrial genome of 24,712 bp was fully assembled, encoding 15 protein-coding genes (PCGs), 2 rRNA genes, and 25 tRNA genes. Furthermore, a complete ribosomal DNA tandem repeat of 7,974 bp was obtained, with a GC content of 51.28%, comprising 18S rDNA (1,795 bp), ITS1 (181 bp), 5.8S rDNA (158 bp), ITS2 (161 bp), 28S rDNA (3,324 bp), and IGS (2,355 bp). This whole-genome dataset provides essential resources for elucidating the genetic basis of insect infection in *Cordyceps* fungi and the molecular mechanisms underlying ecological adaptation, thereby facilitating phylogenetic analyses of *Cordyceps* and supporting applications in entomopathogenic fungal biological control.

## Methods

### Sample collection

**Fig. 1:**
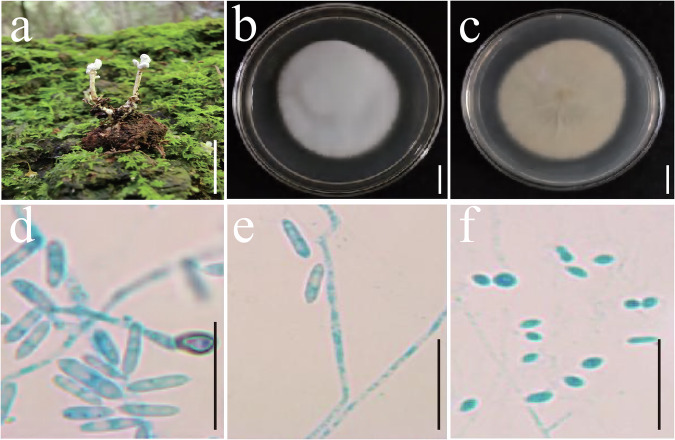
Asexual morphology of GXU-C8616. Note: (**a**) Wild materials; (**b,c**) PDA colonies (obverse and reverse); (**d**–**f**) conidiogenous structures (spore stalks, phialides) and conidia. Scale bars: a = 2 cm; b–c = 1 cm; d–f = 10 μm.

The GXU-8616 strain used in this study was obtained from Maoer Mountain, Guangxi (25°52′59″N, 110°28′59″E) at an elevation of 1,500 m. The strain parasitized *Lepidopteran* pupae, with synnemata emerging from the host’s head. The synnemata were white to off-white, measuring 2–2.5 cm in length, often branched, and produced club-shaped conidial masses (Fig. 1a). When cultured on potato dextrose agar (PDA) at 25 °C for 7 days, the mycelia displayed a cotton-like texture with an off-white reverse side. Colonies reached 5–6 cm in diameter, indicating rapid growth (Fig. 1b,c). Microscopic examination revealed that conidiophores of GXU-8616 originated from hyphae with a length of 12–15 μm; phialides measured 7–9.5 μm in length; and conidia had a diameter of 2–2.5 μm, exhibiting an elliptical or nearly spherical base. The conidia were single-celled, hyaline, smooth, and peanut-shaped (Fig. 1d–f). These morphological traits corresponded to those of *C. cateniannulata* described by Pereira *et al*.^5^, Zhang *et al*.^8^ and Guan *et al*.^11^. The active GXU-8616 strain was cultured on PDA medium and subsequently preserved at the Herbarium of the College of Life Science and Technology, Guangxi University and the designated contact for this material was Luodong Huang (ynhuangld@gxu.edu.cn). Meanwhile, the GXU-8616 strain was deposited in the China Center for Type Culture Collection on May 7, 2025, under accession number CCTCC NO: M 2025989.

### Genomic sequencing

Mycelia cultured for 7 days were harvested, and genomic DNA was extracted using the MiniBEST Universal Genomic DNA Extraction Kit (TaKaRa, Beijing, China) per the supplier’s protocols. For short-read sequencing, paired-end 150 bp sequencing was conducted on the MGISEQ-200 platform at BGI Tech Co., Ltd. (Shenzhen, China), generating 4.93 Gb of raw data (Table S1). For PacBio HiFi sequencing, a 15 kb SMRTbell library was prepared and sequenced on the PacBio Revio platform, producing 1.54 Gb of raw data with an N50 of 19,175 bp. For Hi-C sequencing, libraries were prepared according to the method of Belton *et al*.^12^. Strains were digested with the MboI restriction endonuclease and subsequently subjected to PE150 sequencing on the MGISEQ-200 platform, yielding 8.12 Gb of raw data. For transcriptome sequencing, mycelia were cultured with six distinct carbon sources (20 g/L of potato powder, shrimp shell, oil-tea shell, soybean meal, algae powder, and molasses). Total RNA was extracted from each treatment, and sequencing was performed on Illumina NovaSeq 6000 platform. A total of 37.91 Gb of raw data was procured from the six samples (Table S1) for use in genome structural annotation and gene set refinement.

### Genome prediction and assembly

**Fig. 2:**
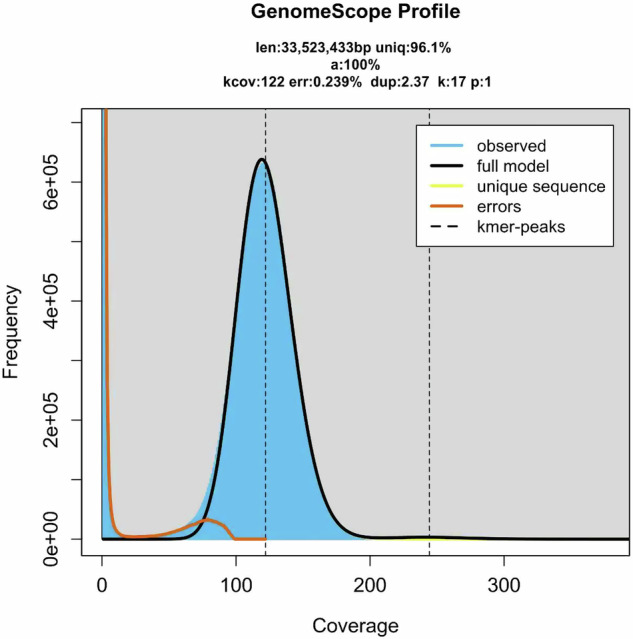
Distribution of 17-mer frequencies in GXU-8616. Note: Frequency distributions of k-mers with different occurrences from two paired-end libraries. K-mer occurrences (x-axis) are plotted against their frequencies (y-axis).

Prior to genome assembly, Second-generation short-read sequencing data were applied to assess the overall characteristics of the GXU-8616 genome. The 17-mer frequency distribution generated by Jellyfish (v2.3.0)^13^ was used as input for GenomeScope (v2.0.0)^14^ to estimate genomic features. The analysis indicated a estimated genome size of 33.5 Mb, with repetitive sequences comprising 2.37% and a heterozygosity rate of 0 (Fig. 2).

**Fig. 3:**
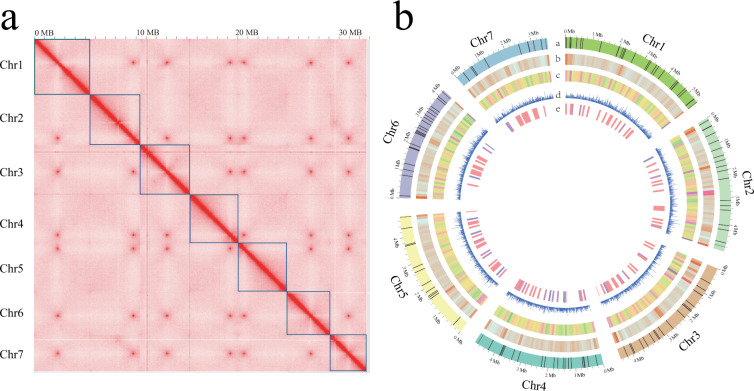
Genome-wide Hi-C heatmap and circular genome landscape of GXU-8616. Note: (**a**) Heatmap of Hi-C interactions among seven pseudochromosomes. (**b**) Circular genome landscape of GXU-8616. From outside to inside: (**a**) chromosome ideograms with lengths, with highlighted lines indicating carbohydrate glycosidase gene loci; (**b**) GC content (blue = high, brown = low); (**c**) gene density (green = high, red = low); (**d**) exon density; (**e**) ncRNA. Chr, chromosome. Sliding windows: (**b,c,e**) 100 kb; (**d**) 1 kb.

Third-generation HiFi data were employed for nuclear genome assembly. An initial draft assembly was produced using hifiasm (v0.19.8)^15^ based on the overlap–layout–consensus approach. The resulting contigs were then subjected to three rounds of polishing with NextPolish (v1.3.1)^16^ using second-generation short-read data. To generate a chromosome-level assembly, Hi-C data were aligned to the contigs of the GXU-8616 genome with Juicer (v1.6)^17^, followed by error correction, contig anchoring, ordering, and orientation with 3D-DNA^18^. Hi-C interaction maps generated by 3D-DNA were subsequently imported into Juicebox (v1.9.8)^19^ for manual refinement of potential assembly errors and the final determination of chromosome boundaries (Fig. 3). Assembly quality was evaluated for completeness using BUSCO (v5.4.7) (-evalue 1e-05)^20^. The final GXU-8616 nuclear genome measured 32.71 Mb, anchored to 7 pseudochromosomes (chromosome-scale scaffolds), with a GC content of 53.82%, an N50 of 4,790,175 bp, and a BUSCO completeness score of 99.0% (fungi_odb10 database) (Table 1). These seven pseudochromosomes represent chromosome-scale assemblies based on Hi-C contact maps and synteny with related *Cordyceps* species, though telomeric repeats were not experimentally confirmed.

**Table 1:** Summary of genome sequencing and assembly of GXU-8616.

Statistic	Values
Assembly size (Mb)	32.71
Number of chromosomes	7
Number of Contigs	7
Number of Scaffolds	7
Scaffold N50(Mb)	4.79
Scaffold max (Mb)	5.46
GC (%)	53.82
Repeat sequences (%)	2.44
Number of non-coding RNA	156
BUSCO genes (%)	99

**Fig. 4:**
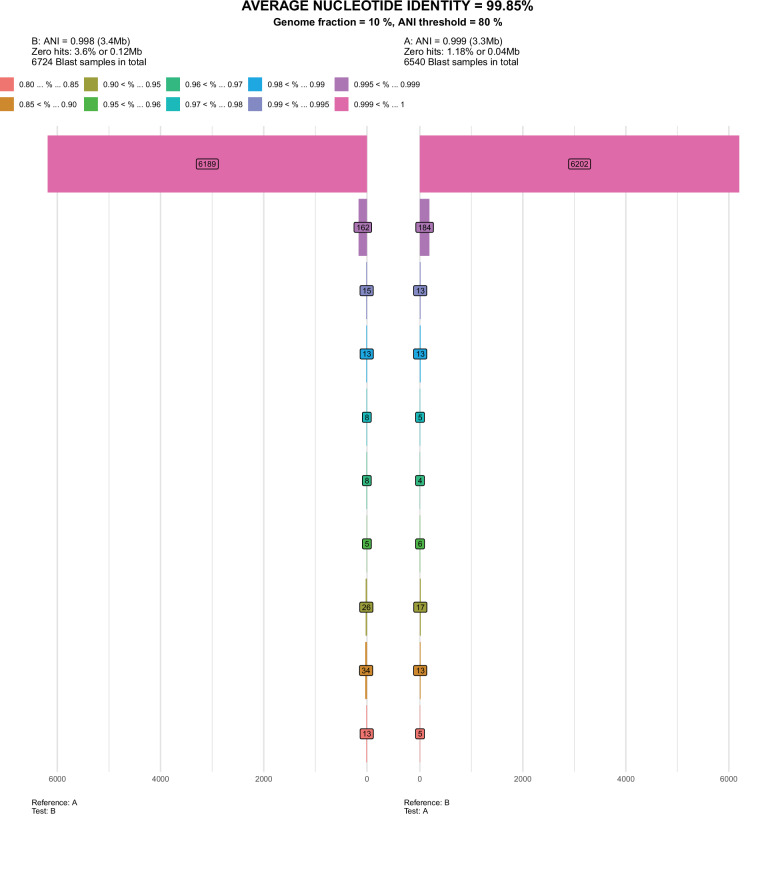
FungANI analysis of GXU-8616 (A) and *Cordyceps cateniannulata* srain FRD24 (B). Note: The final ANI value is shown at the top. Below are the bidirectional ANI values, the percentages of zero hits, estimated sizes of genome-specific regions, and the number of BLAST alignments. The graphic depicts BLAST-detected sequences with color-coded percentages, and boxes show the absolute numbers of BLAST hits within each range.

To resolve the taxonomic placement and phylogenetic relationships of strain GXU-8616, its genome sequence together with six publicly available *C. cateniannulata* genomes from NCBI (Table S3) were analyzed to calculate whole-genome average nucleotide identity (ANI)^21^. ANI was computed using FungANI (v1.3)^22^, which incorporates the NCBI blastn utility (BLAST 2.12.0+) and an internal Python pipeline with multiprocessing support. The six *C. cateniannulata* genomes were iteratively aligned against GXU-8616 using blast comparisons across 1,000 bp sliding windows with 500 bp overlaps, and bidirectional calculations were performed in “fast” mode (10%). Generally, fungal genomes with ANI values > 99.5% are considered conspecific^22^. GXU-8616 displayed the highest ANI value of 99.85% with *C. cateniannulata* strain FRD24^23^, confirming that the two strains belong to the same species (Fig. 4). In contrast, ANI values between GXU-8616 and the other five *C. cateniannulata* strains were 99.16%, 99.12%, 99.09%, 99.06%, and 90.85%, respectively, all falling below the 99.5% threshold (Figures S2–S6). These discrepancies were likely attributable to sequencing errors or variations in genome assembly quality. Therefore, high-quality genome assemblies are essential for reliable microbial species identification.

**Fig. 5:**
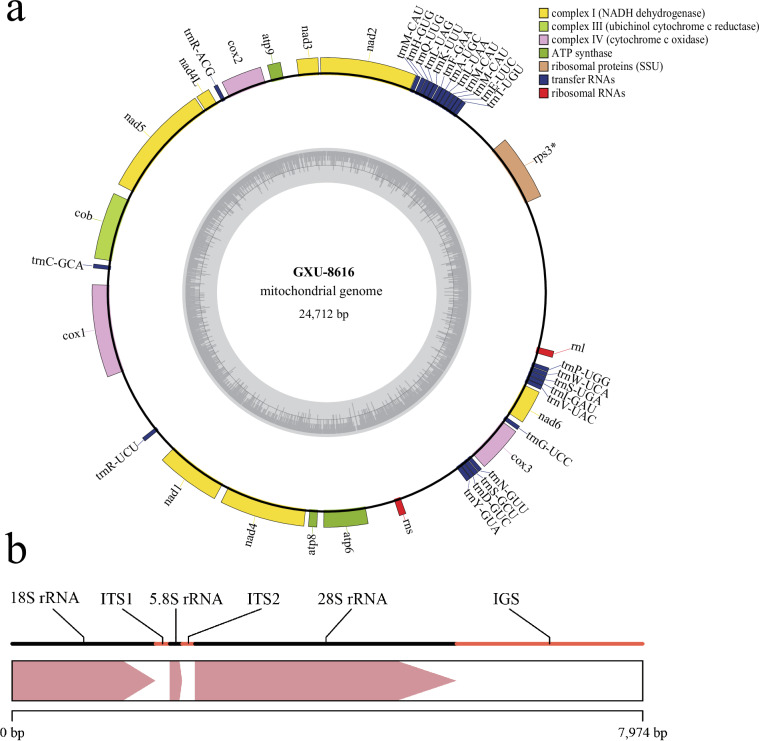
GXU-8616 Mitochondria and Ribosomes Annotation. Note: (**a**) Circular mitochondrial genome map of GXU-8616, with genes categorized by functional groups and color-coded. Differences in color intensity and hue denote gene types. (**b**) Diagram of ribosomal DNA sequence organization: 18S rRNA, ITS1, 5.8S rRNA, ITS2, 28S, and IGS.

Furthermore, second-generation short-read data were applied with GetOrganelle (v1.7.7.0)^24^ to assemble the mitochondrial genome and ribosomal DNA sequences of GXU-8616. The python script was executed using the fungus_mt and fungus_nr databases as references, with parameters set to 30 iterations and candidate K-mer sizes of 55, 75, 95, and 115. Subsequent alignment with third-generation HiFi data, combined with manual inspection, yielded a gap-free circular sequence. The mitochondrial genome of GXU-8616 was identified as a circular DNA molecule of 24,712 bp (Fig. 5a), with sequencing coverage ranging from 509 × to 4,674 × and an average depth of 2,378× (Figure S1). The complete ribosomal DNA repeat was 7,974 bp with a GC content of 51.28%, comprising 18S rDNA (1,795 bp), ITS1 (181 bp), 5.8S rDNA (158 bp), ITS2 (161 bp), 28S rDNA (3,324 bp), and IGS (2,355 bp) (Fig. 5b).

### Annotation

RepeatModeler (v2.0.5)^25^ and RepeatMasker (v4.0.9)^26^ were applied to detect and annotate repetitive elements in the genome. LTR_retriever (v2.9.4)^27^ was specifically used to identify long terminal repeat (LTR) retrotransposons. Repetitive sequences totaled 798,480 bp, representing 2.44% of the GXU-8616 genome, with 225 LTRs accounting for 0.86% (Table S2).

Non-coding RNAs (ncRNAs) were identified using the covariance model implemented in Infernal (v1.1.4)^28^ with the Rfam database (http://xfam.org/). The GXU-8616 genome contained 156 ncRNAs, including 1 ribozyme, 91 tRNAs, 3 other ncRNAs, 38 rRNAs, 19 snRNAs, and 4 sRNAs.

Using the Funannotate (v1.5.0) package (https://funannotate.readthedocs.io/en/latest/index.html), gene prediction was performed with Augustus, SNAP, GlimmerHMM, CodingQuarry, and GeneMark-ES/ET, producing structural annotation files with a BUSCO completeness score of 90.2% (fungi_odb10 database). To enhance annotation accuracy and completeness, manual refinement was conducted with IGV-Gsaman (https://github.com/CJ-Chen/GSAman/releases) in combination with transcriptome data, yielding 10,744 high-confidence genes with an average gene length of 1,598.9 bp and an average coding sequence length of 558.2 bp (Table 2). The corrected gene set achieved a BUSCO completeness of 99.6% (fungi_odb10 database), reflecting a 9.4% improvement relative to pre-correction results and constituting the highest-quality assembly and structural annotation reported for species within the *Cordycipitaceae* family to date.

**Table 2:** Genomic structural annotation statistics for GXU-8616.

Structural Annotation	Values
Protein-coding genesr	10744
Average gene length (bp)	1,598.90
Exons per gene	2.62
Average exon length (bp)	558.9
CDS per gene	2.62
Average CDS length (bp)	558.2
Introns per gene	1.62
Average intron length (bp)	83
Protein sequences (Mb)	5.23
Average protein length (aa)	487

Gene set functional annotation was conducted using the eggNOG platform (v5.0)^29^. Protein sequences were processed through eggNOG-mapper for automated alignment and annotation against prebuilt orthologous group databases, including Kyoto Encyclopedia of Genes and Genomes, Gene Ontology, Clusters of Orthologous Groups, and Protein Families. Annotation was achieved through bidirectional best hit algorithms and orthologous group mapping strategies, with cross-validation across multiple databases to assign gene functions, metabolic pathways, and evolutionary relationships. In total, 8,798 genes were annotated with at least one functional entry, representing 81.89% of the entire geneset (Table 3).

**Table 3:** Nuclear genome geneset functional annotation of GXU-8616.

Functional Annotation	Count	Percentage
Annotation	8798	81.89%
GO	3885	36.16%
EC	2029	18.88%
KEGG	4349	40.48%
CAZy	146	13.59%
COG	8568	79.75%
PFAMs	8443	78.58%
ALL	10744	100%

Mitochondrial genome annotation was initially carried out using the MFannot online tool (megasun.bch.umontreal.ca/apps/mfannot/) with NCBI genetic codon 4. Open reading frames (ORFs) larger than 300 bp were subsequently predicted using the ORF Finder tool in the UGENE platform. tRNA and rRNA genes were identified with tRNAscan-SE 2.0 (http://lowelab.ucsc.edu/tRNAscan-SE/)^30^ and RNAmmer 1.2^31^. Introns were classified using the RNAweasel server (http://megasun.bch.umontreal.ca/cgi-bin/RNAweasel/RNAweaselInterface.pl). All annotation outputs were integrated and verified on the UGENE platform, followed by manual refinement. In total, 42 genes were annotated in the GXU-8616 mitochondrial genome, comprising 15 PCGs, 25 tRNA genes, and 2 rRNA genes (Fig. 5, Table 4).

**Table 4:** Annotated mitochondrial genes of GXU-8616.

Function	Gene
ATP synthase	atp6, atp8, atp9
NADH dehydrogenase	nad1, nad2, nad3, nad4, nad4L, nad5, nad6
Cytochrome c oxidase	cox1, cox2, cox3
Ubichinol cytochrome c reductase	cob
Ribosomal proteins (SSU)	rps3
Other genes	rnl, rns, rns-fragment
Transfer RNAs	trnT-UGU, trnE-UUC, trnM-CAU, trnL-UAA, trnA-UGC, trnF-GAA, trnK-UUU, trnL-UAG, trnQ-UUG, trnH-GUG, trnR-ACG, trnC-GCA, trnR-UCU, trnY-GUA, trnD-GUC, trnS-GCU, trnN-GUU, trnG-UCC, trnV-UAC, trnI-GAU, trnS-UGA, trnW-UCA, trnP-UGG

### Phylogenetics and comparative genomics

**Fig. 6:**
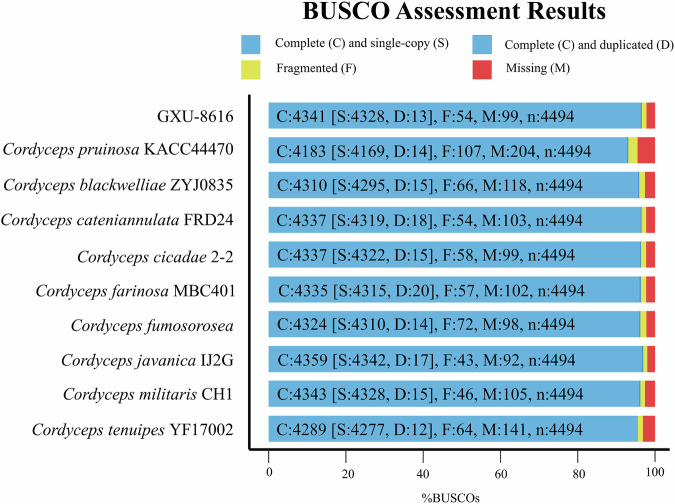
BUSCO assessment of GXU-8616 and representative *Cordyceps* species.

To establish the phylogenetic placement of *C. cateniannulata* strain GXU-8616, 63 nuclear genome datasets from nine *Cordyceps* species were retrieved from the NCBI database (Table S3), and gene completeness was assessed using BUSCO (hypocreales_odb10 database). The BUSCO completeness of GXU-8616 reached 96.6%, markedly higher than that of the six previously reported *C. cateniannulata* strains (Table S3). Among the four *Cordyceps* species with chromosome-level assemblies, the BUSCO of GXU-8616 was only slightly lower than *C. militaris* strain CHI, with no statistically significant difference (Fig. 6), demonstrating that the GXU-8616 genome assembly exhibits high completeness within the genus *Cordyceps*.

**Fig. 7:**
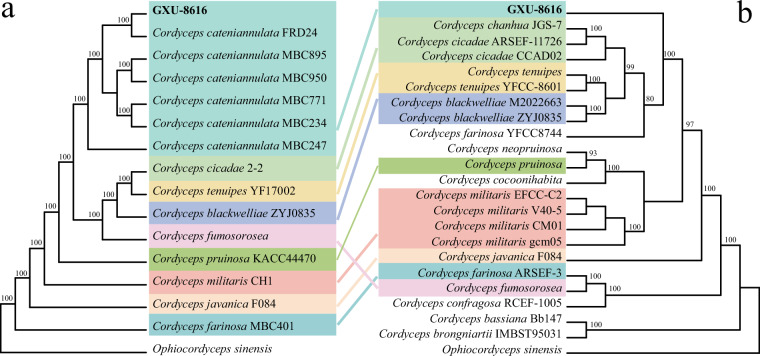
Comparative phylogenetic analyses of mitochondrial and nuclear genomes. Note: (**a**) nuclear genome phylogenetic tree; (**b**) mitochondrial genome phylogenetic tree. Outgroup: *Ophiocordyceps sinensis*.

Five annotated *Cordyceps* genomes from NCBI (accession numbers: GCA_006981975.1, GCA_006981985.1, GCA_008080495.1, GCA_001636725.1, GCA_000225605.1) were integrated with the geneset of GXU-8616, and homologous gene prediction was performed using GeMoMa (v1.9)^32^, generating preliminary annotations for 15 strains across nine *Cordyceps* species (Table S3). Using OrthoFinder v2.3.1^33^, *Ophiocordyceps sinensis* was designated as the outgroup for nuclear gene family clustering, producing 3,177 single-copy orthologous genes. For the mitochondrial phylogeny, complete mitochondrial genomes from 22 strains of 13 *Cordyceps* species in NCBI (Table S4), along with the GXU-8616 mitochondrial genome, were analyzed with *O. sinensis* as the outgroup. HomBlocks collinear block alignment of all mitochondrial genome yielded a 14,834 bp conserved consensus sequence. Nuclear and mitochondrial phylogenetic trees were both constructed using IQ-TREE (v2.1.3)^34^ with 1,000 bootstrap replicates (Fig. 7).

Comparative analysis of phylogenetic trees derived from nuclear and mitochondrial genomes revealed strong concordance in overall topology, with GXU-8616 occupying identical positions in both phylogenies (Fig. 7). At the phylogenetic level, *C. cateniannulata* clustered with *C. cicadae*, *C. tenuipes*, and *C. blackwelliae* to form a closely related clade, whereas *C. pruinosa* and *C. militaris* were positioned in more distant branches. Notably, *C. fumosorosea* displayed marked differences in branch placement between the two genomic datasets.

**Fig. 8:**
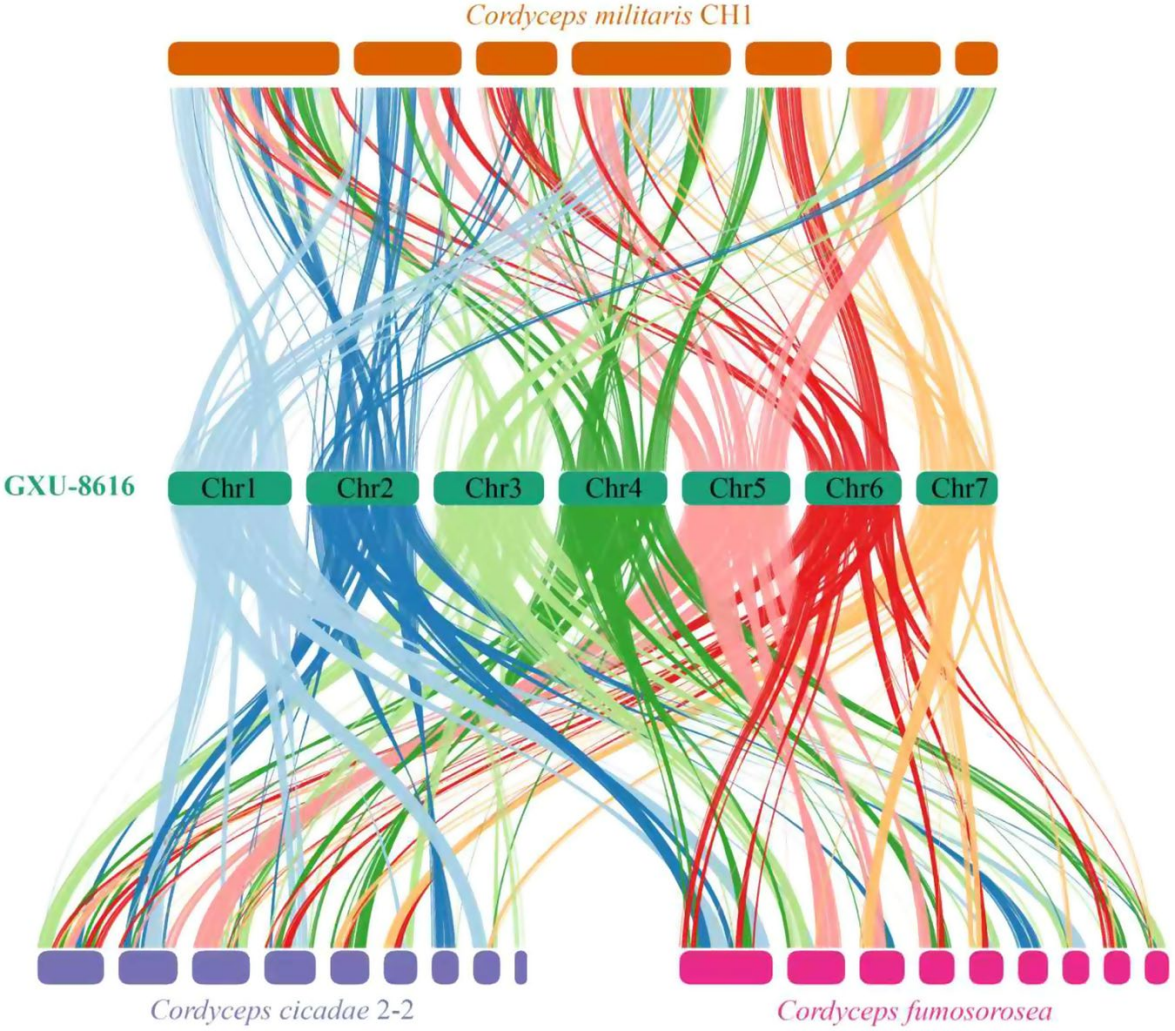
Genome synteny among GXU-8616 and three *Cordyceps* species.

Whole-genome collinearity analysis was conducted to evaluate the structural integrity and chromosome-scale continuity of the *C. cateniannulata* GXU-8616 assembly. The analysis included three additional chromosome-level genomes from the genus *Cordyceps*: *C. militaris* CH1 as the reference, *C. cicadae* 2-2, and *C. fumosorosea* (Fig. 8). Large and continuous syntenic blocks were observed across several chromosomes, supporting the high contiguity and correctness of the assembly. Chromosomes 5 and 7 showed strong collinearity with their counterparts in the reference genomes, indicating conserved genomic structure. In contrast, chromosomes 2 and 4 exhibited more fragmented syntenic alignments and complex rearrangement patterns relative to *C. militaris* CH1. These differences are consistent with lineage-specific structural variation and do not result from assembly errors.

These results provide essential resources for future studies on the genetic basis of insect infection in *Cordyceps* species and the molecular mechanisms underlying ecological adaptation, thereby facilitating phylogenetic analyses of *Cordyceps* fungi and supporting applications in entomopathogenic fungal biocontrol.

## Data Records

The genomic data of *C. cateniannulata* have been deposited in NCBI under the following accession numbers: PRJNA1268321^35^ (BioProject) and SAMN48746374^36^ (BioSample). Raw sequencing data, including BGI, PacBio, Hi-C, and transcriptome reads, are available in the NCBI Sequence Read Archive with accession numbers SRR34289951-SRR34289959^37–45^. The assembled nuclear genome has been archived in China National Center for Bioinformation (CNCB) under accession number PRJCA046752^46^. Concurrently, it has been submitted to the NCBI under BioProject PRJNA1268321. The genome assembly is available under the Whole Genome Shotgun (WGS) project JBRIOS000000000.1 (accession: JBRIOS0100000001-JBRIOS010000007)^47^. The assembled mitochondrial genome and ribosomal sequences have been archived in NCBI, with the accession numbers PV93651536^48^ and PV91676737^49^ respectively.

## Technical Validation

Genome assembly quality assessment: BUSCO completeness scores were 99.0% (fungi_odb10) and 96.6% (hypocreales_odb10). Together with the 7 chromosome N50 value of 4.79 Mb, these metrics confirm the high-quality assembly of the GXU-8616 genome with respect to completeness and chromosome-level continuity. Additionally, a circular mitochondrial genome of 24,712 bp and a circular ribosomal DNA sequence of 7,974 bp were fully assembled for GXU-8616.

Gene annotation reliability validation: The 10,744 protein-coding gene of geneset exhibited a BUSCO completeness of 99.6% (fungi_odb10), with 81.89% of genes functionally annotated, demonstrating that the GXU-8616 genome annotation possesses both structural integrity and functional coverage.

## Supplementary information

Supplementary information

Supplementary Table S3

Supplementary Table S4

## Data Availability

BGI, PacBio, Hi-C, and transcriptome raw data have been deposited in the NCBI Sequence Read Archive with accession numbers SRR34289951-SRR34289959^36–44^. The nuclear genome assembly and annotation results of *C. cateniannulata* has been submitted to China National Center for Bioinformation (CNCB) with the accession number GWHGQKB00000000.1^50^. Concurrently, the nuclear genome assembly is available under the Whole Genome Shotgun (WGS) project JBRIOS000000000.1 (accession: JBRIOS0100000001-JBRIOS010000007)^47^ Additionally, the mitochondrial genome and ribosomal DNA repeat sequences are publicly accessible via the NCBI GenBank with the accession numbers PV936515^46^ and PV916767^47^ respectively.

## Code availability

The software programs utilized in this study were referenced in the Methods section. If detailed parameters for the software were not specified, default parameters were used.
