# Supplementary material for: A chromosome-scale nuclear genome and complete mitogenome of the bio-control fungus Cordyceps cateniannulata
Source: Sci Data. 2026 Apr 14;13:871. doi: 10.1038/s41597-026-07231-1 (PMC13254215; doi:10.1038/s41597-026-07231-1)
Supplement: Supplementary file 1 — Supplementary information [file 41597_2026_7231_MOESM1_ESM.pdf]

Supplementary information

contents

Table S1. Statistics of sequencing data used for genome assembly. 2

Table S2. Annotated repetitive elements in the GXU-8616 genome. 3

Figure S1. Sequencing depth and coverage map of the GXU-8616 mitogenome. 4

Figure S2. FungANI analysis of GXU-8616 with *Cordyceps cateniannulata* strain MBC950 5

Figure S3. FungANI analysis of GXU-8616 with *Cordyceps cateniannulata* strain MBC895 6

Figure S4. FungANI analysis of GXU-8616 with *Cordyceps cateniannulata* strain MBC234 7

Figure S5. FungANI analysis of GXU-8616 with *Cordyceps cateniannulata* strain MBC771 8

Figure S6. FungANI analysis of GXU-8616 with *Cordyceps cateniannulata* strain MBC247 9

Table S1. Statistics of sequencing data used for genome assembly.

Sequencing Platform	Reads	Total Bases	GC Content (%)
Short reads	32,855,256	4,928,288,400	52.90
Hi-C	79,223,320	1,188,349,800	53.32
PacBio HiFi	147,159	2,771,173,571	53.19
RNA-Seq(all)	372,565,560	55,884,834,000	55.83

Table S2. Annotated repetitive elements in the GXU-8616 genome.

Category	Number of elements	Total Bases	GC Content (%)
SINEs	0	0	0
LINEs	0	0	0
LTR	225	281910 bp	0.86 %
DNA transposons	94	52445 bp	0.16 %
Unclassified	180	42132 bp	0.13 %
Small RNA	50	41242 bp	0.13 %
Simple repeats	8143	339839 bp	1.04 %
Low complexity	843	40912 bp	0.13 %
total	9535	798480 bp	2.44 %

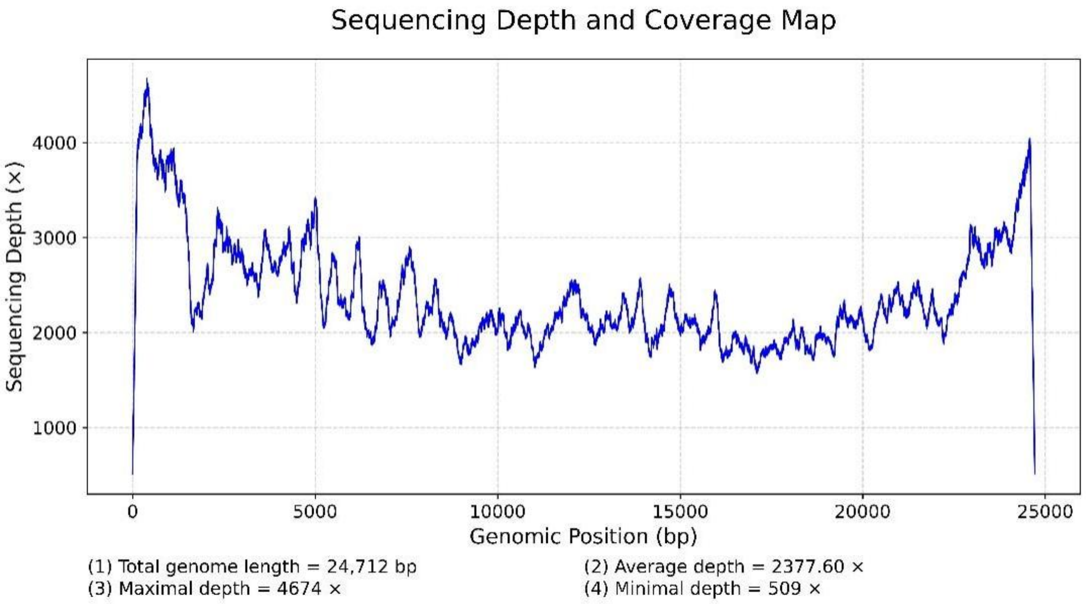

Figure S1. Sequencing depth and coverage map of the GXU-8616 mitogenome.

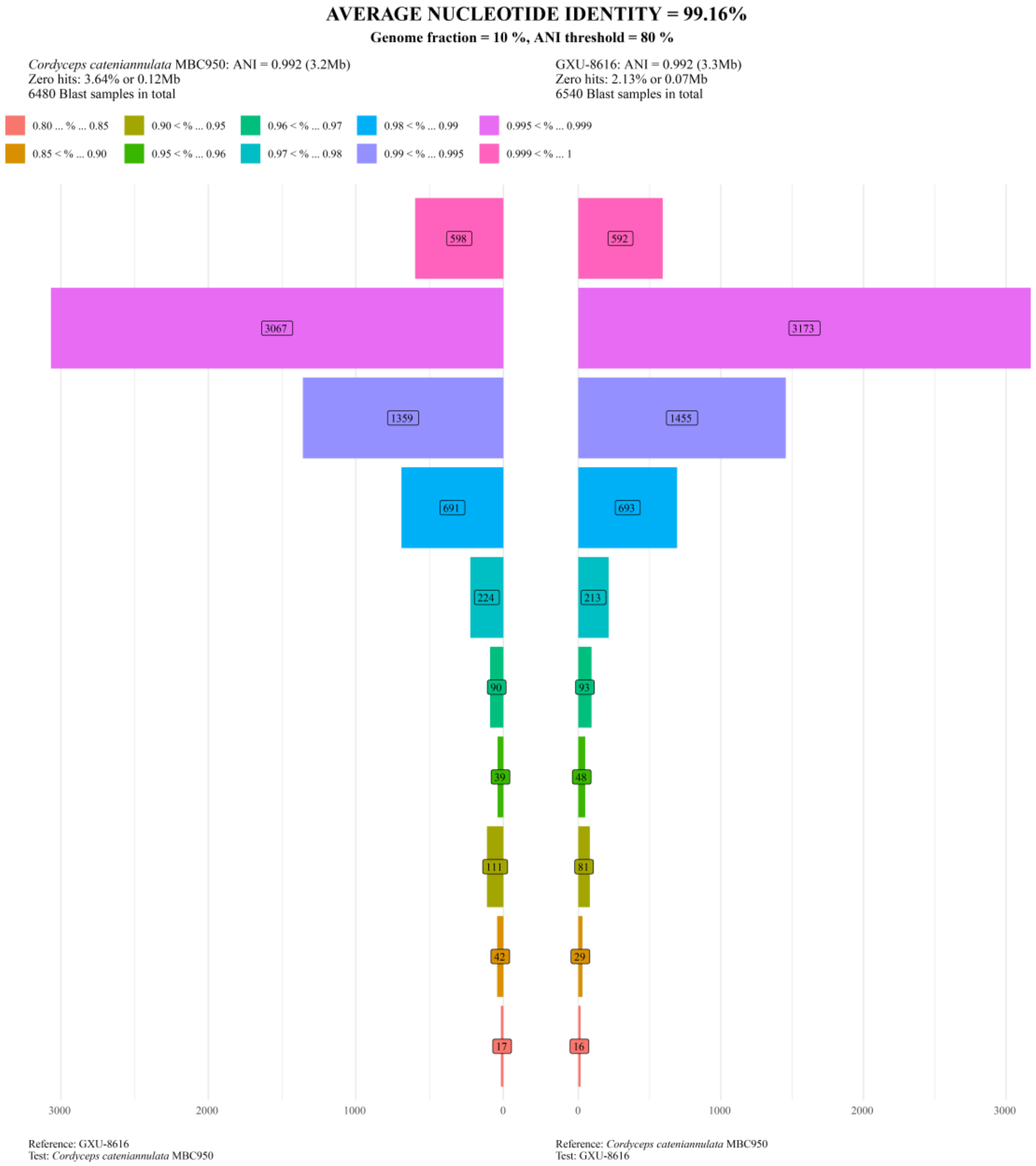

Figure S2. FungANI analysis of GXU-8616 with *Cordyceps cateniannulata* strain MBC950.

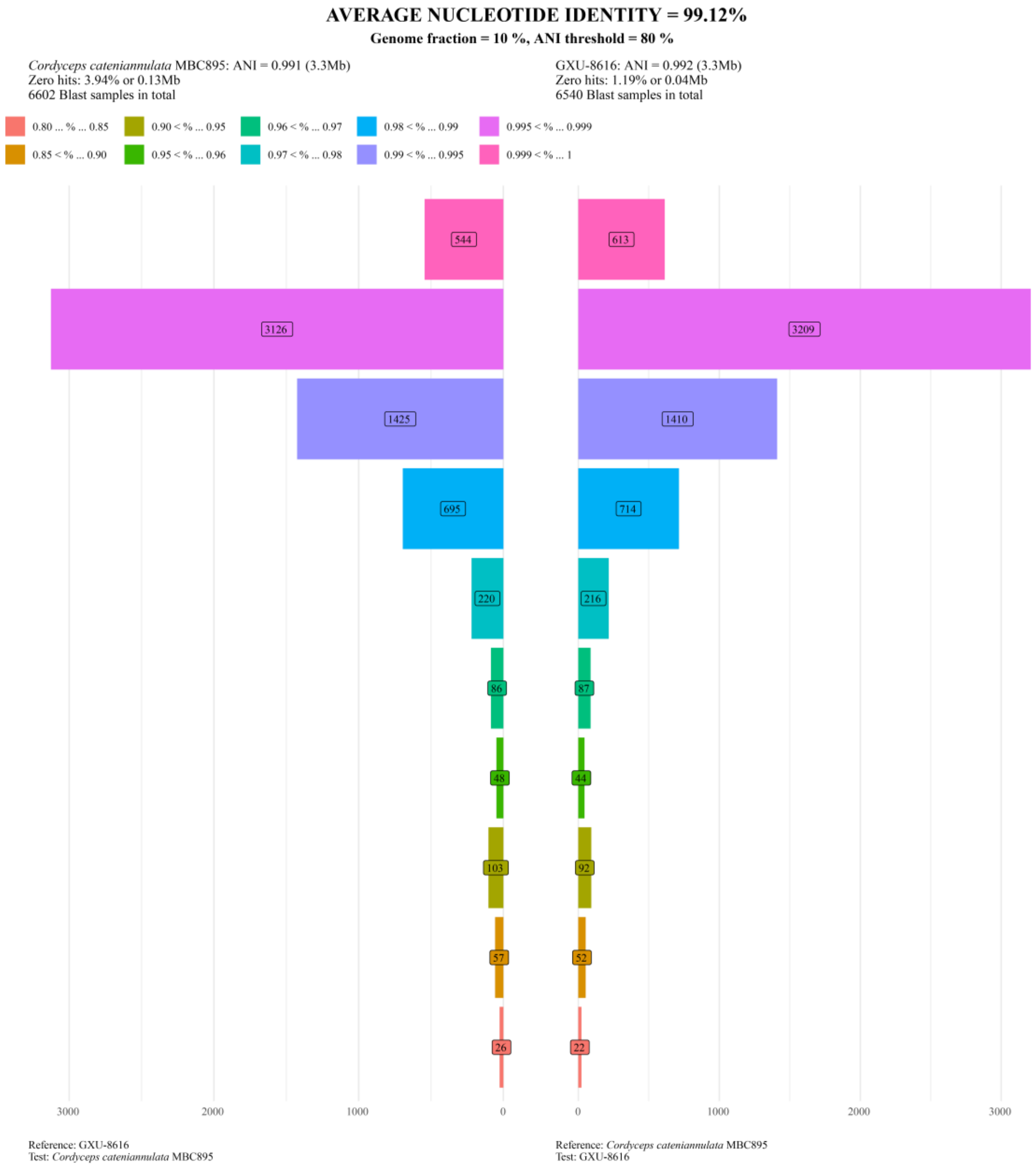

Figure S3. FungANI analysis of GXU-8616 with *Cordyceps cateniannulata* strain MBC895.

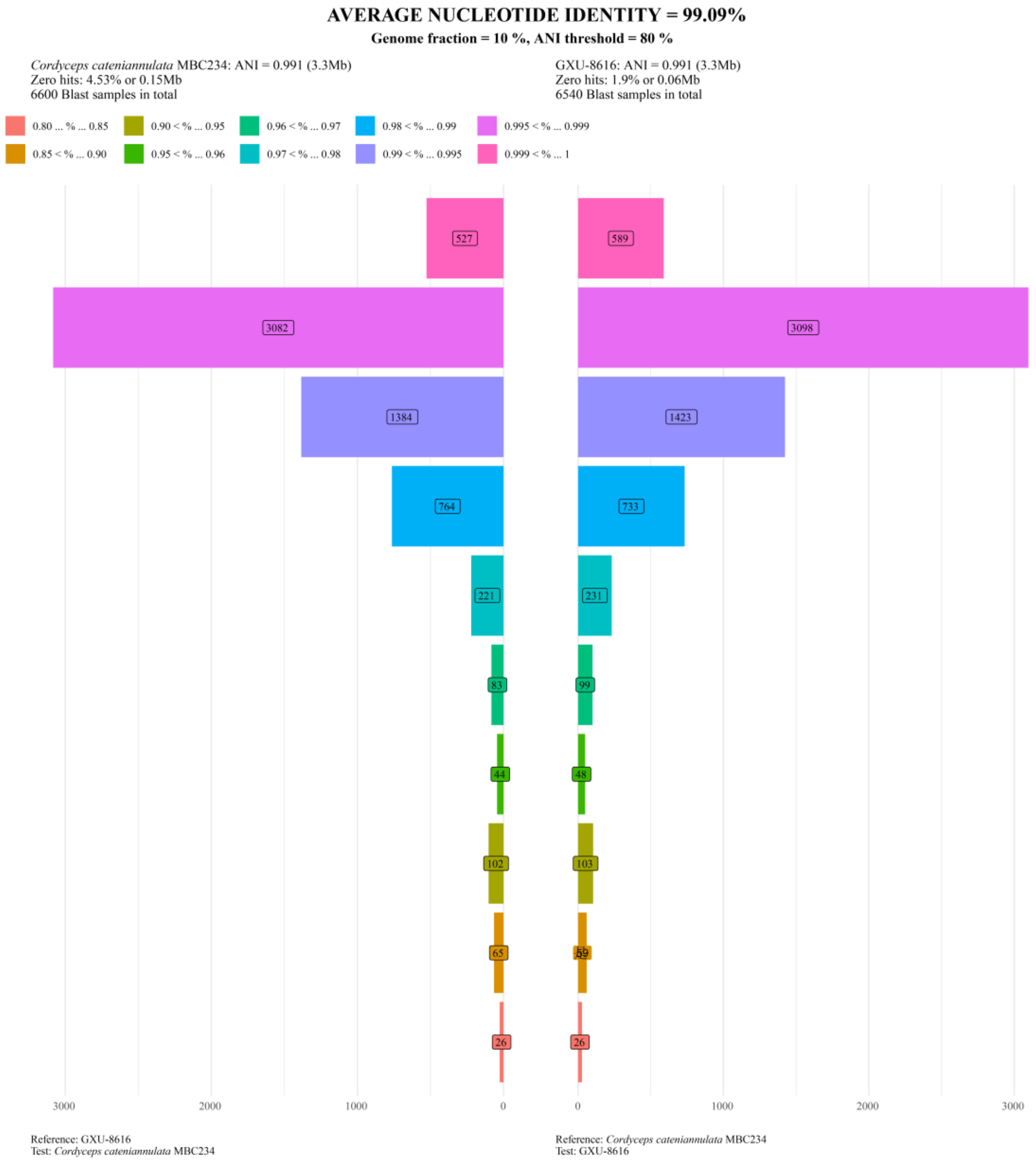

Figure S4. FungANI analysis of GXU-8616 with *Cordyceps cateniannulata* strain MBC234.

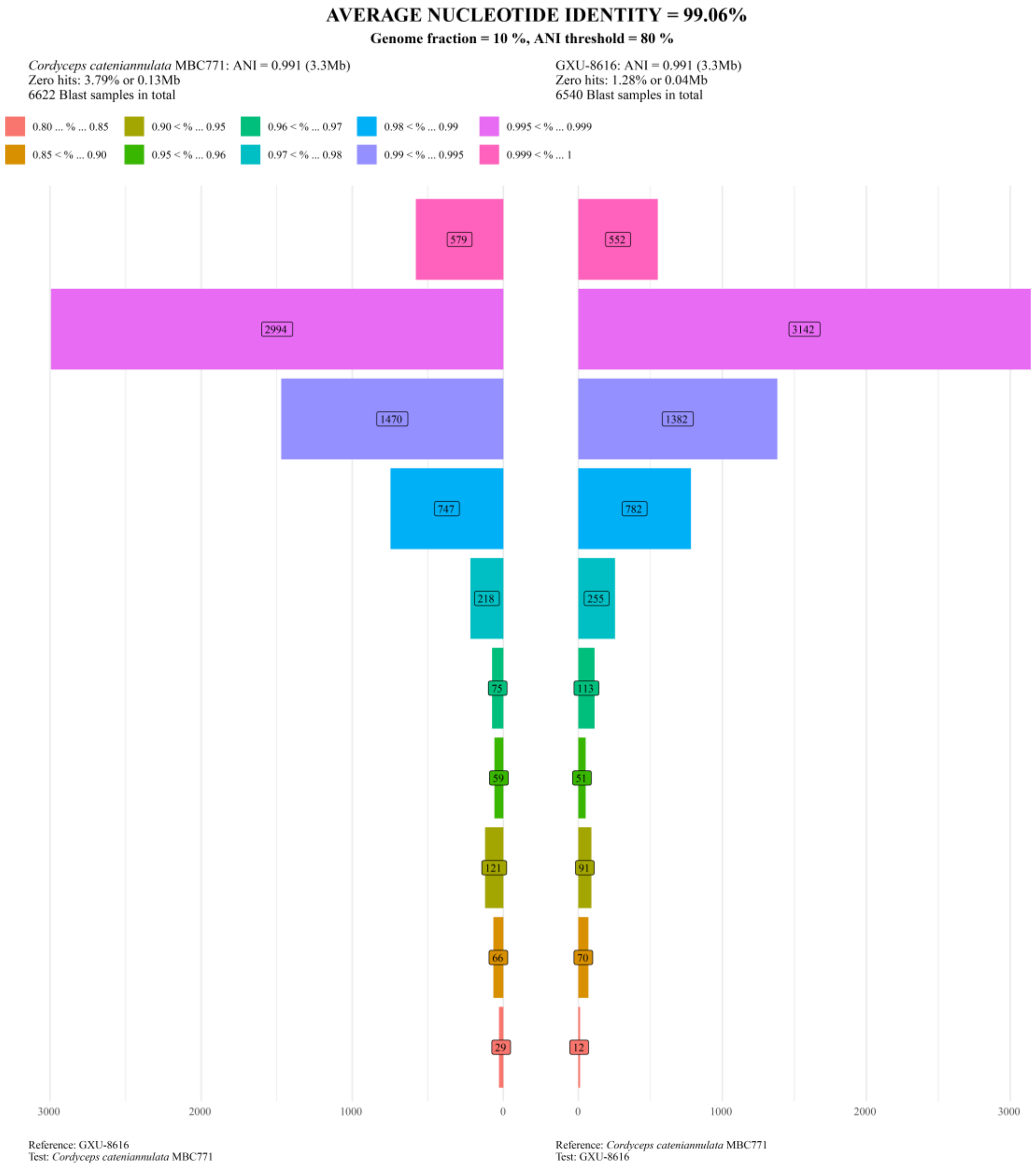

Figure S5. FungANI analysis of GXU-8616 with *Cordyceps cateniannulata* strain MBC771.

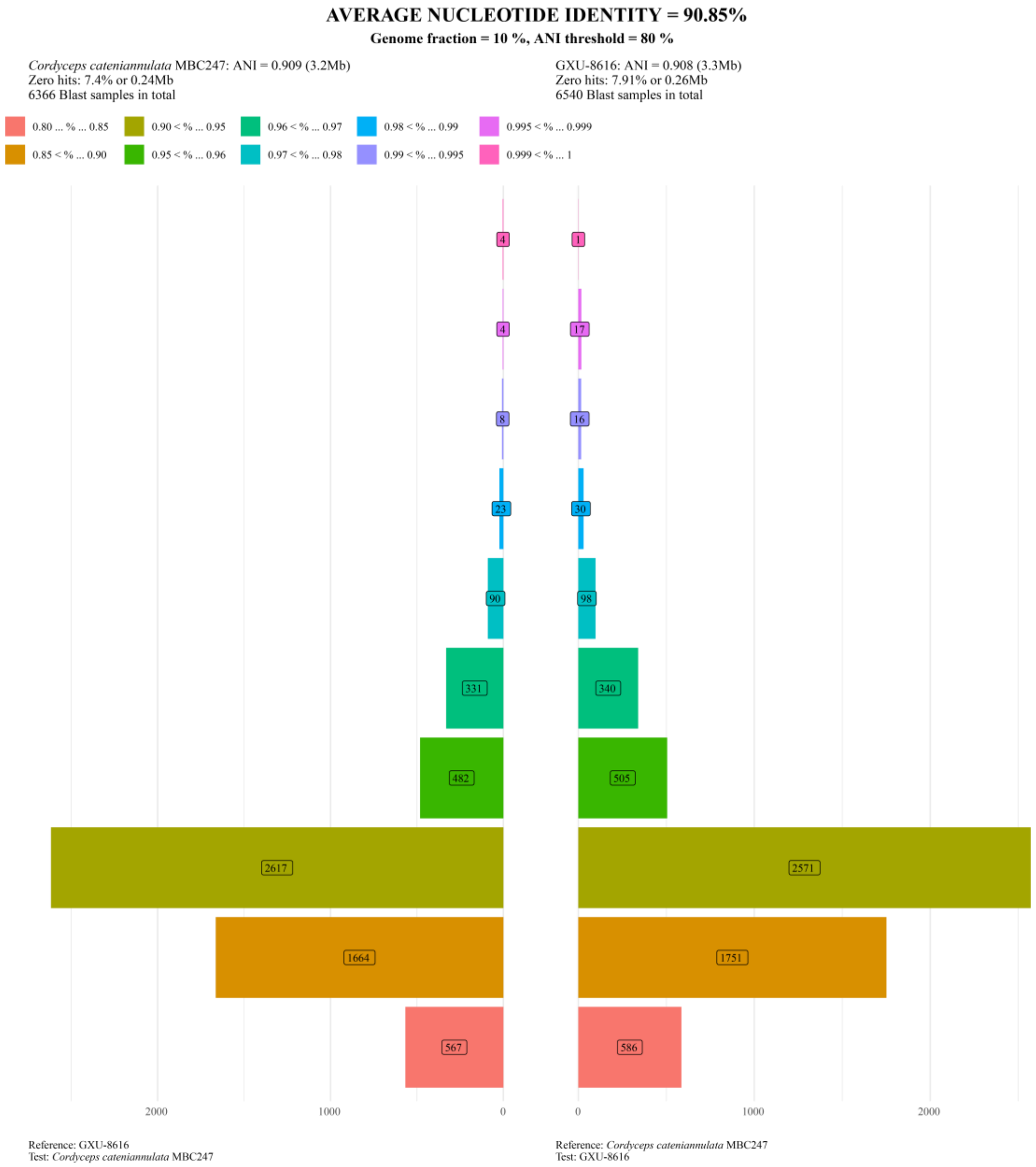

Figure S6. FungANI analysis of GXU-8616 with *Cordyceps cateniannulata* strain MBC247.
